# 肺鳞癌分子靶向治疗的研究现状

**DOI:** 10.3779/j.issn.1009-3419.2014.08.07

**Published:** 2014-08-20

**Authors:** 亚楠 李, 云芝 周, 洪武 王

**Affiliations:** 100028 北京，北京煤炭总医院肿瘤内科 Department of Medical Oncology, China Meitan General Hospital, Beijing 100028, China

**Keywords:** 肺鳞癌, 分子靶点, 分子特征改变, 靶向治疗, Squamous-cell lung cancer, Molecular targets, Molecular alterations, Targeted therapy

## Abstract

肺癌是目前世界上发病率和死亡率最高的恶性肿瘤之一, 其中肺鳞癌(squamous-cell lung cancer, SQCLC)是一种最常见的肺癌类型, 经手术、放化疗等综合治疗后, 其5年生存率仍低于15%。而目前分子靶向治疗在肺鳞癌治疗中发挥重要作用, 迫切需要对其进行更深入的研究。肺鳞癌治疗的分子靶向药物主要以表皮生长因子受体(epidermal growth factor receptor, EGFR)、磷脂酰肌醇-3-激酶催化亚单位α(phosphoin-3-kinase catalytic alpha polypeptide, PIK3CA)、成纤维细胞生长因子受体1(fibroblast growth factor receptor 1, FGFR1)、盘状结构域受体2(discoidin domain receptor 2, DDR2)、第10号染色体缺失的磷酸酶及张力蛋白同源的基因(phosphatase and tensin homolog deleted on chromosome ten, PTEN)、BRAF、MET、胰岛素样生长因子1受体(insulin-like growth factor 1 receptor, IGF-1R)等为靶点的药物, 有的靶向药物正在研发, 有的靶向药物已进入临床试验。随着近年来肺鳞癌分子靶向治疗的相关研究, 分析靶向治疗在肺鳞癌中的发展及在提高患者生存率、改善生存质量等研究方面取得的实质性进展, 使肺鳞癌的个体化靶向治疗成为可能。

## Abstract (en)

Lung cancer is one of the world's highest morbidity and mortality disease in malignant tumors currently.Squamous-cell lung cancer (SQCLC) is one of the most prevalent subtypes of lung cancer worldwide, after surgery, radiotherapy, chemotherapy and other comprehensive treatment, its 5-year survival rate is still below 15%.The current molecular targeted therapy plays an important role in the treatment of SQCLC, an urgent need to be more in-depth study.SQCLC molecular targeted therapy mainly epidermal growth factor receptor (EGFR), phosphoin-3-kinase catalytic alpha polypeptide (PIK3CA), fibroblast growth factor receptor 1 (FGFR1), discoidin domain receptor 2 (DDR2), phosphatase and tensin homolog deleted on chromosome ten (PTEN), BRAF, MET, insulin-like growth factor 1 receptor (IGF-1R) and other as the target of the drug, some targeted drugs are being developed, and some targeted drugs have entered clinical trials.In recent years, with studies molecular targeted therapy in SQCLC, analysis of the development and trgeted therapy achieved substantial progress in improving the survival rate of SQCLC, and other research to improve the quality of life, make is possible to individualized targeted therapy of SQCLC.

肺癌是当今世界最常见的人类恶性肿瘤之一, 其死亡率居癌症相关死因的首位。诊断的肺癌患者中约80%-85%为非小细胞肺癌(non-small cell lung cancer, NSCLC), 其中肺鳞癌(squamous-cell lung cancer, SQCLC)约占NSCLC的20%-30%^[1, 2]^, 属于一种常见的肺癌病理类型^[3]^。SQCLC治疗除传统手术及放化疗外, 靶向治疗成为其目前治疗的重要手段。随着表皮生长因子受体酪氨酸激酶受体抑制剂(epidermal growth factor receptor tyrosine kinase inhibitor, EGFR-TKI)问世, 给肺腺癌患者带来巨大获益后, 肺腺癌的各种靶向药物也已相继进入临床, 其治疗效果、生存期得到明显改善。相比肺腺癌, SQCLC的研究较滞后, 仍无有效的靶向药物指导临床实践^[4]^, 原因可能是由于缺乏对其生物学特征的了解。随着对基因学的深入研究为认识SQCLC分子生物学特征提供了可能。在SQCLC发生发展过程中, 相关分子靶点表皮生长因子受体(epidermal growth factor receptor, EGFR)、磷脂酰肌醇-3-激酶催化亚单位α(phosphoin-3-kinase catalytic alpha polypeptide, PIK3CA)、成纤维细胞生长因子受体1(fibroblast growth factor receptor 1, FGFR1)、盘状结构域受体2(discoidin domain receptor 2, DDR2)、第10号染色体缺失的磷酸酶及张力蛋白同源的基因(phosphatase and tensin homolog deleted on chromosome ten, PTEN)、BRAF、MET、胰岛素样生长因子1受体(insulin-like growth factor 1 receptor, IGF-1R)等发挥着重要作用。本文就SQCLC潜在分子靶点EGFR、PIK3CA、FGFR1、DDR2、PTEN、BRAF、MET、IGF-1R结构特点、生物学特征改变及靶向药物治疗的最新研究进展综述如下。

## 1 EGFR

EGFR是原癌基因*c-erbB1*的表达产物, 是一个具有酪氨酸激酶活性的糖蛋白受体, 属于表皮生长因子受体(HER)家族成员之一, 该家族包括HER1(ErbB-1)、HER2/c-neu(ErbB-2)、HER3(ErbB-3)和HER4(ErbB-4)。*EGFR*基因位于第七号染色体短臂上(7q12), 长约118 kb, 由28个外显子组成。编码的EGFR是分子量为170 kDa的跨膜糖蛋白, 编码的蛋白由1, 186个氨基酸残基组成。EGFR位于细胞膜的表面, 靠与配体结合来激活, 目前发现的与EGFR胞外区结合的配体有:表皮生长因子(epidermal growth factor, EGF)、转化生长因子α(transforming growth factor-α, TGF-α)、B细胞生长因子(B-cell growth factor, BCGF)、表皮调节素(epiregulin, EPR)。EGFR活化后可激活下游多条细胞信号转导途径, 激活可能导致细胞的分化、增殖、浸润以及新血管的发生^[5, 6]^, 其高表达还可导致组织癌变, 促进肿瘤细胞生长、粘附及远处转移, 与预后差有关。

*EGFR*在SQCLC中的突变率文献报道各不相同, *EGFR*基因突变率在东亚裔肺腺癌人群中为30%-40%, 但在同地区其他组织类型的NSCLC(包括SQCLC)中则较低^[7, 8]^。Mu等^[9]^报道SQCLC的*EGFR*突变率为5.9%(6/102), 而Lai等^[10]^报道282例SQCLC中有41例*EGFR*突变, 突变率为14.5%。Hata等^[11]^研究认为, 纯SQCLC无*EGFR*突变。然而有些学者认为, EGFR在SQCLC中有突变是由于肺SQCLC中混有肺腺癌成分所致。通过王碧波等^[12]^认为*EGFR*突变确实发生在肺SQCLC患者中, EGFR是SQCLC的驱动基因之一。在临床研究和实践中, 肺腺癌患者*EGFR*突变率明显比SQCLC的高, 且大部分EGFR-TKI对肺腺癌患者有益, *EGFR*突变的SQCLC患者是否也具有肺腺癌突变者同样的EGFR-TKI治疗疗效, 目前缺乏大样本的研究^[13]^报道, 因此需大样本的证实才能得出准确的结论。Shukuya^[14]^等临床研究对33例非腺癌患者进行分析, 其中27例为鳞癌, 3例为腺鳞癌, 21例患者发生*EGFR*突变, 证实*EGFR*突变的SQCLC患者应用EGFR-TKI治疗的有效率、疾病控制率、中位无进展生存期(27%、67%-70%和3个月)明显低于*EGFR*突变的肺腺癌患者(66%、92%-93%和9.4个月)。SQCLC *EGFR*突变者临床获益程度也明显低于*EGFR*突变的肺腺癌患者, 之所以远远低于肺腺癌患者, 可能原因是与EGFR下游多条信号通路异常活化有关。如在肺癌中可检测到*PIK3CA*突变和拷贝数增加, 且在SQCLC患者中出现的频率明显高于肺腺癌患者^[15]^。有关SQCLC分子靶向药物EGFR-TKI—厄洛替尼的相关研究报道极少, 但根据前瞻性研究和有关文献证实, SQCLC厄洛替尼的治疗疗效及患者的耐受程度似乎好于吉非替尼, 这是否与在常规治疗下厄洛替尼处于高的血药浓度及体外研究显示其对部分野生型肿瘤细胞有效相关, 尚未明确仍需进一步研究。还有学者认为SQCLC厄洛替尼治疗有效者也可能是通过*EGFR*突变以外的机制调节介导的。另外有研究^[13]^报道, 约30% SQCLC有*EGFR*扩增, 故基因的高拷贝数可能是预测SQCLC EGFR-TKI治疗疗效的一个指标。因此随着分子生物学和基因工程技术的飞速发展, 利用其对大量EGFR-TKI治疗有效的SQCLC患者进行全方面、深层次的研究、分析, 有可能发现EGFR潜在的药物治疗新靶点^[13]^。

## 2 PIK3CA

*PIK3CA*基因是由利用原位杂交技术(*in situ* hybridization, ISH)检测到的一种癌基因、一种脂质激酶编码基因, 还是逆转录病毒v-p3k癌基因在细胞内的同系物, 其长34 kb, 定位于3q26.32, 包含20个外显子, 编码1, 068种氨基酸, 该氨基酸产生一组长124 kDa的蛋白。*PIK3CA*基因是由一个85 kDa调节亚基和一个110 kDa催化亚基组成, 能编码Ⅰ类特异性磷酸化磷脂酰肌醇-3-激酶(phosphatidylino sitol 3-kinases, PI3Ks)的p110催化亚单位。在生长因子的作用下, p85与酪氨酸激酶受体结合解除了p85对p110a的抑制, 从而使PIP2磷酸化生成PIP3^[16]^。PIP3作为第二信使可激活AKT, PI3K/AKT细胞信号转导通路是调节细胞功能的重要途径, PI3Ks通过参与PI3K/AKT信号通路调节细胞的增殖、粘附、迁移和凋亡^[17, 18]^。*PIK3CA*突变主要发生在螺旋区和激酶区, 且大部分集中在外显子9和20。PIK3CA可在结肠癌、乳癌、肝癌、胃癌、肺癌等不同类型肿瘤中发生体细胞突变, 且在结肠癌、乳癌、肝癌、胃癌突变率比较高, 而在NSCLC中相对少见^[19, 20]^。

Okudela等^[20]^运用荧光原位杂交(fluorescence *in situ* hybridization, FISH)法检测*PIK3CA*在肺癌中的突变率低为4.2%。Kawano和他的同事^[21]^调查确认日本肺癌患者*PIK3CA*的突变率也较低为3.6%, 并且证明其在鳞癌的突变率为5.6%, 在腺癌突变的发生率仅为1.5%, 由此可见*PIK3CA*在鳞癌突变率明显高于腺癌。而*PIK3CA*在SQCLC中的扩增率较突变率高, Ji等^[22]^用聚合酶链反应(polymerase chain reaction, PCR)法证实近一半中国SQCLC患者有*PIK3CA*扩增。目前已有针对*PIK3CA*突变的靶向药物进入肺癌早期临床研究中, *PIK3CA*发生体细胞突变的活性可以被PI3K特异性抑制剂LY294002所抑制, 其也可抑制NSCLC细胞系生长且还与放化疗具有协同作用^[23]^。而PI3K特异性抑制剂BKM120的Ⅰ期临床实验结果也证明一半以上的患者疾病处于稳定期, 且患者一般耐受性好。目前BKM120的Ⅱ期正在进行有针对转移性NSCLC的临床试验, 所有患者均接受P13K/AKT信号通路的检测, 或可通过检测*PIK3CA*基因突变明确其变异与BKM120治疗疗效的相关性^[24]^。另外, 通过检测发现SQCLC *EGFR*敏感性突变的患者, EGFR-TKI的治疗疗效明显低于突变的肺腺癌患者, 可能与脂质激酶活性增强及EGFR下游P13K/AKT细胞信号转导通路异常激活密切相关。大量研究^[25]^结果表明*PIK3CA*基因突变见于具有*EGFR*基因突变的肿瘤患者, *PIK3CA*突变与*EGFR*突变为非排他性, 如两者共同存在突变则可导致耐药, 影响EGFR-TKI的治疗疗效^[25]^, 对此还需进一步进行大规模、多方面、深入性的临床研究。

## 3 FGFR1

FGFR1是FGFR家族成员之一^[17]^, 是一类具有自身磷酸化活性的跨膜酪氨酸激酶受体。该基因位于染色体8p12, 其在细胞增殖、分化、抗凋亡、迁移以及血管生成中起重要作用。FGFR1酪氨酸激酶家族包括FGFR1、FGFR2、FGFR3和FGFR4, 通过扩增、突变或易位从而导致细胞发生癌变^[26]^。*FGFR1*基因扩增是SQCLC最常见的改变之一。Weiss等^[27]^研究的结果也显示, *FGFR1*基因扩增主要见于SQCLC, SQCLC常见于吸烟患者, 由此*FGFR1*基因扩增的患者倾向于不良的生存预后。

Zhang等^[28]^分析报道中国人*FGFR1*基因在SQCLC中的扩增率高于肺腺癌患者, 与Heist等^[29]^报道的美国、德国患者结果一致。Dutt等^[30]^分析证实了57例SQCLC样本中, 有*FGFR1*扩增的SQCLC为21%, 而腺癌仅占3%。Schildhaus等^[31]^研究报道402例NSCLC组织中FGFR1的水平, 发现其中有20%-30%的SQCLC患者存在扩增, 而肺腺癌中未见扩增。中外研究均证实, SQCLC中*FGFR1*扩增率明显高于肺腺癌, 且在亚欧人群中不存在明显的种族差异。Weiss等^[27]^在SQCLC的细胞中首次发现FGFR1激活可引起肿瘤细胞的生长, 特异性阻断其激活, 能使肿瘤细胞靶病灶明显缩小。FGFR1过表达与吸烟的鳞癌患者有关, 提示烟草中可能有破坏该蛋白质的编码基因, 该编码基因与SQCLC的发生发展密切相关。还通过对155例SQCLC患者中进行单核苷酸多态性(single nucleotide polymorphism, SNP)分析, 发现15例存在*FGFR-1*基因缺陷, 其中11例有吸烟史, 余4例吸烟状态具体不详。而借助FISH的方法在其他153例SQCLC标本中进行检测, 得出22%存在*FGFR1*扩增^[18]^。相比较鳞癌患者, 非鳞癌患者基因缺陷发生率低。肺癌FGFR1抑制剂是PD173074, 通过PD173074的作用进行分析, 发现PD173074能抑制肿瘤细胞的生长并导致其死亡。进一步研究动物试验表明, FGFR1抑制剂PD173074治疗存在*FGFR1*基因缺陷的SQCLC患者, 能使患者从中获益。以FGFR1为靶点的抑制剂BIBF1120、BGJ398、TK1258和E3180等研究正在进行之中, 其中进行的一项Ⅱ期临床试验研究^[32]^结果显示, 以化疗失败后的晚期NSCLC患者, 接受BIBF1120治疗后中位无进展生存期可长达5个月, 通过对其分析显示鳞癌与非鳞癌患者可同样获益。

## 4 DDR2

*DDR2*基因定位于染色体1q23.3, 是一种可以和胶原蛋白结合的受体型酪氨酸蛋白激酶。在细胞调节中发挥重要作用, 可调节细胞的增殖、分化、凋亡。DDR2配体为纤维型胶原, 通过其与受体结合调节细胞外重建机制、可诱导细胞内DDR2磷酸化还可诱导EGFR下游多条信号转导^[24]^。DDR2的激活也与肿瘤细胞的生长和转移有关, 其活性形式主要是体细胞突变, 而扩增相对比较少见。

Hammerman等^[33]^研究报道290例鳞癌组织标本*DDR2*总体突变率为3.8%, 277例SQCLC组织样本突变率为3.2%。通过对*DDR2*突变率与临床病理特征的关系进行分析, 发现*DDR2*突变与患者的年龄、性别、吸烟情况等特征无明显相关性。同时大量学者还发现, DDR2是SQCLC潜在的药物靶向治疗位点。EGFR-TKI—达沙替尼可抑制*DDR2*突变的SQCLC患者细胞的生长, 因此可认为*DDR2*突变与达沙替尼具有相关性。在对DDR2进行临床研究测试发现, *DDR2*突变可以被达沙替尼联合厄洛替尼抑制剂所抑制。如1例接受达沙替尼和厄洛替尼联合治疗的*EGFR*野生型的SQCLC患者, 在治疗后稳定期可长达14个月之久。如果大样本量研究能够证实, *DDR2*突变对达沙替尼疗效具有预测作用^[34, 35]^, 则可为SQCLC分子靶向治疗带来新的突破, 更可进一步为中晚期SQCLC尤其是为耐化疗药及化疗失败的患者提供更全面、更合理的治疗方案。

## 5 PTEN

PTEN定位于染色体10q23.3区, 全长200 kb, 由9个外显子和8个内含子组成, 其cDNA含有1, 209个核苷酸组成的开放阅读框架, 编码着由403个氨基酸组成、分子量为47, 000的蛋白质。PTEN蛋白中与抑癌功能相关的结构由氨基端(N端)磷酸酯酶结构域、脂质结合的C2结构域和羧基端(C端)结构域这3个区域共同组成^[36]^。在PTEN蛋白的氨基酸序列中, N端与细胞骨架张力蛋白(tensin)、神经触突泡转运相关辅助蛋白(auxilin)具有高度同源性。其中辅助蛋白与神经突触小泡的运输密切相关, 张力蛋白参与细胞的聚集与粘附。脂质结合C2结构域能以Ca^2+^非依赖性方式使PTEN与细胞膜磷脂结合, 参与PTEN催化结构域在细胞膜的正确有效定位和体内细胞的信号转导^[37]^。羧基端结构域包括两个PEST序列和尾去的一个PDZ结构域^[38]^, PEST序列酪氨酸、丝氨酸/苏氨酸残基磷酸化和PDZ结构域蛋白质之间的蛋白-蛋白的作用对调节其自身的稳定性和酶活性有重要作用。PTEN可使丝氨酸、苏氨酸和酪氨酸残基去磷酸化, 拮抗由蛋白酪氨酸激酶(PTK)介导的信号传导, 同时作为酯性磷酸酶、磷酸酰肌醇-3, 4, 5三磷酸酯(PIP3)及负性调节1-磷酸酰肌醇-3-激酶(PI3K)的信号传导通路^[39, 40]^。

*PTEN*基因失活可表现为突变、缺失及PTEN mRNA或蛋白的低表达、失表达、甚至不表达。PTEN具有不稳定性, 其失活在NSCLC中广泛存在。Tang等^[41]^报道发现, 46.1%的NSCLC患者存在PTEN缺失, 其中鳞癌高达52.9%。PTEN缺失也可引起PI3K/AKT等多条下游信号通路的异常调节, 促进肿瘤细胞增殖、迁移、粘附, 抑制其凋亡, 并介导肿瘤患者对治疗的耐药。目前尚无特异性针对*PTEN*基因的靶向治疗药物, 但由于PTEN是PI3K/AKT信号通路的负性调节因子, 故其能阻断PI3K/AKT信号通路, 而起到抗肿瘤治疗的作用, 因此主要靶向治疗药物均特异性作用于下游AKT靶点。据文献报道, *PTEN*基因突变与AKT抑制剂有紧密的关联性, 因此AKT抑制剂可治疗*PTEN*突变的肿瘤患者。目前正在研发的AKT抑制剂有GDC-0068、MK-2206等, 其中GDC-0068的研究正处于NSCLC治疗的Ⅰ期临床试验, 而MK-2206则已进入Ⅱ期临床试验^[24]^。

## 6 BRAF

*BRAF*基因首先是在人类尤文氏瘤中发现并克隆确认, 是一个活性的DNA序列, 与CRAF和ARAF具有很高的同源性, 因此称其为BRAF。BRAF是RAF家族成员之一, 该家族还包括ARAF、CRAF^[42]^。*BRAF*基因位于人类染色体7q34, 编码着783个氨基酸的蛋白, 其是最为关键的激活因子。BRAF蛋白是RAS-丝裂原激活蛋白激酶(MAPK)信号通路中一种重要的丝氨酸/苏氨酸蛋白激酶, 参与调控细胞生长、分化和凋亡。发生突变后的BRAF能持续激活MAPK通路, 激活后可导致MEK/ERK信号通路过度紊乱, 紊乱后的MEK/ERK与细胞核内的转录因子相结合, 激活下游多种因子, 从而导致细胞的增殖、恶变。

目前*BRAF*基因突变最主要有2种类型:约20%的突变发生于外显子11, 80%发生于外显子15, 即酶功能域的V600E。*BRAF*基因的突变在黑色素瘤是最早发现的也是最常见的, 其次是甲状腺癌, 在直肠癌、卵巢癌、淋巴瘤、肝癌及肺癌中发生率低。约3%的SQCLC中存在*BRAF*突变, 且突变的类型以发生在外显子11为主, 通过此检测研究可能为临床治疗带来新的思路^[43]^。BRAF抑制剂已研制出很多, 有索拉菲尼、PLX4032、GSK2118436等, 其中索拉菲尼已被批准用于肝癌、肾癌等多种肿瘤, 属于多个受体激酶抑制剂, 因此缺乏特异性。以PLX4032为代表的BRAF抑制剂在发生*V600E*突变的黑色素瘤临床试验中取得了明显的治疗疗效, GSK2118436选择性抑制剂在对胶质瘤的研究中显示了明显的抗肿瘤活性, 而目前针对NSCLC的Ⅱ期临床研究正在进行试验中^[44, 45]^。

## 7 MET

MET属于能编码肝细胞生长因子(hepatocyte growth factor, HGF)酪氨酸激酶受体(receptor tyrosine kinases, RTKs)超家族成员之一。MET是一种具有自主磷酸化活性的跨膜受体, 还是一种酪氨酸激酶受体^[46]^。MET位于人类染色体7q31区, 其持续激活后可促进肿瘤细胞增殖、分化、抗凋亡。与此同时, MET扩增也是肿瘤细胞对吉非替尼产生耐药的原因之一^[47]^。

以前普遍认为MET在肺腺癌和SQCLC两种NSCLC中的扩增水平无明显差别, 而目前认为其实在肺癌组织中MET的真正扩增是比较少见的。Go等^[48]^在97例SQCLC中检测到6%左右的MET的真实扩增, 而肺腺癌却未检测到。以MET为靶点的小分子抑制剂有PF-02341066、GSK13630889、XL184等均已相继进入临床试验中。Hellerstedt等^[49]^调查的一项以肺癌晚期(Ⅳ期)为主的Ⅱ期临床试验, 约30%的肺癌患者病理类型为鳞癌, 接受Cabozantinib(XL184)治疗的患者中约有65%出现肿瘤消退现象。

## 8 IGF-1R

IGF-1R是一种跨膜的酪氨酸蛋白受体, 属于跨膜酪氨酸受体家族, 对细胞的增殖、分化及凋亡具有非常重要的调控作用。IGF-1R可与IGF家族中的IGF-Ⅰ、IGF-Ⅱ、胰岛素3个配体结合, 而与IGF-Ⅰ结合的亲和力最高, 结合后可激活酪氨酸激酶, 启动下游一系列细胞信号转导通路, 参与肿瘤的发生发展, 促进细胞增殖分化、抑制细胞凋亡^[50]^。*IGF-1R*基因位于染色体15q26, 能促进G_1_期至S期细胞的增殖, 抑制肿瘤细胞的凋亡。

Nakagawa等^[51]^发现, IGF-1R在SQCLC中的表达率明显高于肺腺癌表达率(46.8% *vs* 23.6%)。亦有研究结果也证实, IGF-1R表达在SQCLC中较其他类型NSCLC更为多见, 在SQCLC中占41.3%, 在其他类型NSCLC中共占34.2%。IGF-1R抑制剂有以IGF-1R为靶点的单克隆抗体figitumumab(CP-751871)、以OSI-906为代表的IGF-1R小分子抑制剂。前者已进入临床研究, Karp等^[52]^进行的一项临床随机对照试验显示, 接受卡铂+紫杉醇化疗合并应用figitumumab的SQCLC患者客观有效率为约达80%左右。后者抑制剂也已进入临床试验阶段^[44]^。

近年来随着基因工程技术的迅速发展及靶向药物的不断研发, EGFR-TKI开启了NSCLC靶向治疗的新方向, NSCLC的治疗已逐步进入基于分子标志物的个体化治疗时代。但靶向药物治疗受益的人群往往是肺腺癌患者, SQCLC的相关研究比较落后, 仍缺乏有效的治疗手段和临床有效的靶向药物。目前除EGFR、PIK3CA、PTEN、FGFR1、DDR2、BRAF、MET、IGF-1R是SQCLC的靶向治疗位点, 尚还有EGFRvⅢ、K-ras、TP53、SOX2等。PI3K/AKT等在内的通路也值得关注, 上述SQCLC的这些靶向治疗位点、通路已逐渐成为热点, 正在被众多学者进行深入的研究。而相关的有效的临床靶向治疗药物有的正在研发, 有的靶向药物已进入临床试验阶段。通过不断地对SQCLC分子生物学特征的认识, 更多的SQCLC分子靶点将被不断的发现, 相关的个体化靶向治疗、靶向药物也将取得深入的发展。相信通过对SQCLC分子生物学特征基础的掌握、采用分子标志物进行大样本前瞻性患者筛选的临床研究以及分子特征的检测受到足够重视, 并且综合考虑组织学分型、分子分型, 不但有助于尽快明确肿瘤的生物学特性, 而且可以为SQCLC患者选择更加合理的、个体化治疗方案。肺腺癌通常为单个驱动基因的改变, 而SQCLC可表现为数个驱动基因或几条信号通路同时发生变化, 提示联合靶向治疗可能对SQCLC的治疗更加有效。
